# Effect of ‘Q’ Ratio on Texture Evolution of Ti-3Al-2.5V Alloy Tube during Rolling

**DOI:** 10.3390/ma15030817

**Published:** 2022-01-21

**Authors:** Qi Yang, Songxiao Hui, Wenjun Ye, Zhe Xu, Chun Dai, Yuan Lin

**Affiliations:** 1 State Key Laboratory of Non-Ferrous Metals and Processes, GRINM Group Co., Ltd., Beijing 100088, China; yangqi@baoti.com (Q.Y.); huisx@grinm.com (S.H.); 2 GRIMAT Engineering Institute Co., Ltd., Beijing 101407, China; 3 General Research Institute for Nonferrous Metals, Beijing 100088, China; 4 BAOTI Group Co., Ltd., Baoji 721014, China; xuzhe@baoti.com (Z.X.); daichun@baoti.com (C.D.); linyuan@baoti.com (Y.L.)

**Keywords:** Ti-3Al-2.5V alloy, texture, ‘Q’ ratio, strain vector

## Abstract

Ti-3Al-2.5V alloy was usually the α phase of HCP structure at room temperature which had obvious anisotropy. During tube rolling, α grain would be influenced by stress-strain state, deformation amount, ‘Q’ ratio to result the preferred orientation and formed texture. In order to obtain radial texture tube by rolling and improve the service quality of tube in the pipeline system, Φ25 mm Ti-3Al-2.5V alloy tubes was selected as billet for the experiment, and four kinds of tubes with outer diameter of Φ16mm was produced by single pass cold rolling with ‘Q’ ratios ranging from 0.65 to 2.0. The effect of ‘Q’ ratio on the texture of Ti-3Al-2.5V tube was studied. The result indicted that the initial texture of the tube is radial-circumferential equally distributed, and the radial basal texture enhances gradually with increasing ‘Q’ ratio. Since the angle between the C-axis of grain and the radial axis of RD decreases, the C-axis of grain distributes to the radial direction, and the more grain orientation from {112X} pyramidal to {0001} basal plane. The different ‘Q’ ratio would lead to different strain along the radial direction, circumferential direction, axial direction, thus affected the crystal orientation and distribution during tube rolling deformation. When ‘Q’ > 1, the tube mainly produced radial basal texture. By comparison with ‘Q’ < 1, the tube mainly produced circumferential basal texture. As a result, when the initial texture of the tube is radial-circumferential equally distributed, the ideal radial texture of the tube can be obtained by choosing rolling process with ‘Q’ > 2.0.

## 1. Introduction

In the selection of materials for aerospace applications, Ti-6Al-4V alloy is the most common titanium alloy. However, the plasticity of Ti-6Al-4V alloy is not enough to produce tube successfully. Low-alloying Ti-3Al-2.5V has high strength, high corrosion resistance and great cold deformation ability, which was used to produce tubes and was applied to aircraft hydraulic systems [1]. In the pilger cold-rolling process, because of the strong anisotropy of α phase with hcp structure, it is easy to form texture. If the texture can be optimized or controlled by a reasonable process so that the basal texture could mainly be distributed along the radial direction of the tube, and the ability of resisting the thinning of the tube wall can be greatly enhanced when the hydraulic thin-walled tubing is subjected to the action of internal pressure, so as to improve the service reliability of the tube.

In recent years, the evolution of the texture of hexagonal metals has been extensively studied, and the effect of deformation and heat treatment on the texture has been deeply revealed [2,3,4,5,6]. The preferred orientation of grains usually follows a specific crystallographic direction and is closely related to the stress direction in the deformation. When producing titanium and zirconium tube, the loading direction are mainly influenced by ‘Q’ ratio, and ‘Q’ ratio is the ratio of natural logarithm for wall reduction rate to diameter reduction rate. Q > 1 means that the tube is mainly characterized by wall reduction deformation, while Q < 1 means that the tube is mainly characterized by diameter reduction deformation [7]. In the study of zirconium alloy tube for nuclear reactors, in order to make zirconium hydride have a specific orientation, Tenckhoff et al. [8,9], Kallstrom et al. [10], Linga Mutry et al. [7] have systematically studied the texture, formation mechanism and anisotropy of zirconium alloy tube. It was considered that the ‘Q’ ratio of rolling is the key parameter affecting the deformation texture of tube. ‘Q’ ratio determines that the effective compressive stress is along the radial or circumferential direction of the tube, and the grain basal pole is mainly parallel to the direction of effective compressive stress. At present, there are relatively few studies on the texture of Ti-3Al-2.5V alloy tube. Zhan et al. [11] mainly studied the effect of tube drawing on the texture. Wagner et al. [12], Linga Murty et al. [13], Zhou et al. [14] studied the effect of annealing temperature on the texture and anisotropy of tube. Zhang et al. [15,16], Yang et al. [17] mainly studied the texture characterization and EBSD analysis of tube. For the two-roll or three-roll rolling process of tube, Huang et al. [18,19], Li et al. [20,21], Wu et al. [22], Nie et al. [23] conducted finite element simulation, and discussed the influence of die and rolling parameters on the texture of tube. In the influence of rolling process on the texture of Ti-3Al-2.5V titanium alloy tube. Rees et al. [24] briefly described how to control the texture of tube by rolling process, and also believed that the texture was determined by the ratio of wall and diameter reduction during rolling process. Chen et al. [25] analyzed the evolution mechanism of texture under several three-roll rolling processes and believed that the type of texture was related to the change of slip mechanism during rolling. In the initial stage of rolling, it is mainly (1010} prism slip. With the increase of deformation, {0001) basal slip instead of prism slip become the main deformation mode. When the deformation further increases, pyramid slip instead of basal slip become the main deformation mode. Hong et al. [26] made a preliminary discussion and research on the influence of ‘Q’ ratio of rolling on texture of tube under three-roll condition, when ‘Q’ > 1, the tube has radial texture, and when ‘Q’ < 1, the tube has circumferential texture. However, these studies were not systematic and in-depth. The effect of rolled ‘Q’ ratio on the texture of Ti-3Al-2.5V tube has not been fully revealed. Now, it is generally believed that the texture of titanium and zirconium alloy tube is mainly controlled by the strain during rolling process [10,27]. Different ‘Q’ ratio would lead to different strain along the radial, circumferential and axial direction, thus affecting the basal pole orientation of the hexagonal crystal.

Ti-3Al-2.5V tube applied to aircraft hydraulic systems was mainly produced by cold rolling using two-roll pilger mill, and the actual cold rolling deformation is more complicated than three-roll tube cold rolling processes. At present, the effect of rolling deformation and ‘Q’ ratio and other technological parameters on the formation and evolution of tube texture is not clear, so it is necessary to study the effect of rolling ‘Q’ ratio on the texture of tube.

## 2. Experimental Method

### 2.1. Experimental Materials

The Φ25 × 2.8 mm Ti-3Al-2.5V alloy tube produced by Baoji Titanium Industry Co., Ltd. (Baoji, China). was selected as experimental material, which was the intermediate tube billet processed by Ti-3Al-2.5V titanium alloy ingot after forging, extrusion, multi-pass cold rolling and recrystallization annealing. Material composition is shown in Table 1. Four kinds of tubes with outer diameter of Φ16 mm were rolled by single pass cold rolling of Φ25 × 2.8 mm tube billet with different ‘Q’ ratio ranged from 0.65 to 2.0. The ‘Q’ ratio was selected according to the parameter range which can be provided by existing dies. The cold-rolled tube (CR) was annealed in vacuum with 490 °C/3 h to obtain the annealed tube. The pressure was 1.3 × 10^−3^ Pa during vacuum annealing treatment. Specific tube sizes and deformation parameters is shown in Table 2. The outer diameter and wall thickness of the tube are the average of 8 equal points on the cross section of the tube and the test position avoided the head and tail of the tube. The deformation amount ε is calculated by the cross-sectional area method, and the ‘Q’ ratio is the ratio of natural logarithm for the wall reduction rate to the diameter reduction rate.

### 2.2. Specimen Preparation and Measurement Techniques

The microstructure and texture samples of the tube were sampled by the expansion method. First, removing tube inner and outer surface and retain the intermediate ring by mechanical method. then, the circular tube thin slices with thickness of 0.05 mm were obtained by pickling thinning. The thin slices were cut and expanded elastically and pasted on the surface of the prefabricated cuboid copper substrate to keep the circumferential and axial directions of the tube thin slices consistent with the length and width of the copper block, respectively. Finally, the flat tube was successively ground by mechanical grinding with 1000#, 2000# and 4000# grit SiC papers, and the sample was prepared by electrolytic polishing at a voltage of 70 V for 15 s. HF:HNO_3_:H_2_O solution with the ratio of 1:3:7 was selected for pickling thinning, and 5% perchloric acid and 95% glacial acetic acid solution was selected for electrolytic polishing.

**Figure 1:**
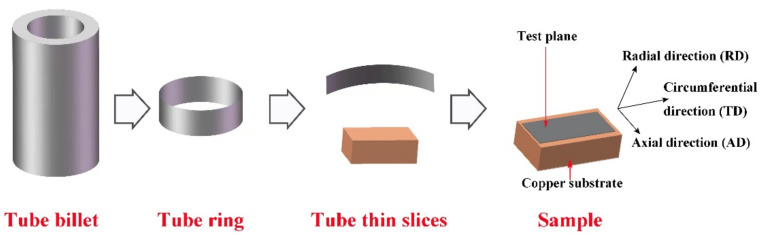
Schematic diagram of specimen preparation.

JEOL F7900 scanning electron microscope and Oxford EBSD instrument were used for texture and microstructure testing. The test plane was the radial plane of the tube, with AD, TD and RD, respectively, representing axial, circumferential and radial directions of the tube, as shown in Figure 1. Amplification factor of 250×, step size 0.5 were selected for testing parameters to ensure that the number of grains counted exceeds 1000. The grain orientation data of the tube was derived by the OIM software supporting EBSD instrument in the form of orientation imaging, pole figures, inverse pole figures and so on.

## 3. Experimental Results and Discussion

### 3.1. The Microstructure

**Figure 2:**
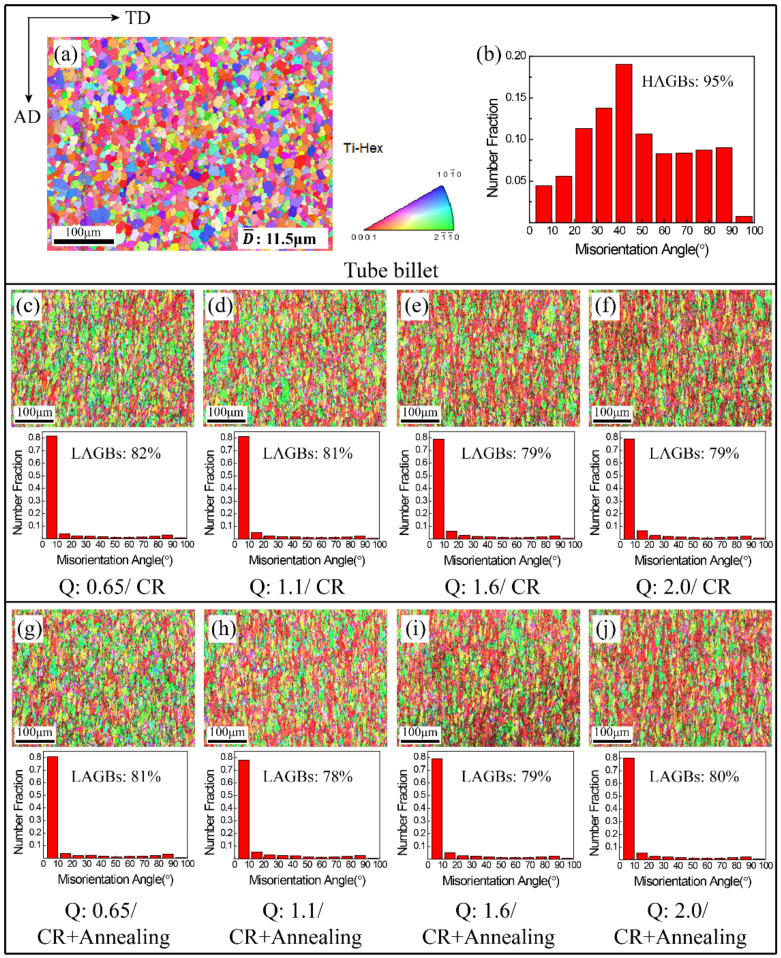
Orientation image and misorientation distribution of grain boundary of tubes. (**a**) Orientation image of tube billet; (**b**) misorientation distribution of grain boundary of tube billet; (**c**–**f**) cold rolled tubes; (**g**–**j**) annealed tube.

Φ16 mm tubes with different wall thicknesses were obtained by rolling Φ25 mm tube billets with ‘Q’ ratios of 0.65, 1.1, 1.6 and 2.0 in a single pass. The microstructure of tube billets, cold-rolled tube (CR) and annealing tube is shown in Figure 2. Figure 2a is the orientation image of the radial plane of the tube billet. Before rolling, the tube billet was dominated by equiaxed α grains of recrystallized, most of which <0001>∥RD, with an average grain size of about 11.5 μm. Figure 2b is the diagram of grain boundary misorientation distribution of the tube billet. The high angle grain boundary (HAGBs) with grain boundary misorientation > 10° is the main distribution of the original tube billet, with HAGBs accounting for more than 95%, indicating that the original microstructure is completely recrystallized.

As shown in Figure 2c–f, through a single pass cold rolling, the original tube grains along the axial elongated, forming tiny uniform deformation structure. As the ‘Q’ ratio increases, the tube wall thickness thinning increases, tube deformation increased from 53% to 70% (Table 1), the length-width ratio of grain is gradually increasing also. When the ‘Q’ ratio is 2.0, the aspect ratio reaches 5–6. The tube after stress-relieved annealing (Figure 2g–j) has basically the same microstructure and morphology as the cold-rolled tube. Figure 2c–j shows the grain boundary misorientation distribution of the tube after rolling and annealing. For the tube after single pass cold rolling, the grain boundary misorientation is mainly distributed in low angle grain boundaries (LAGBs) of 0–10°, accounting for about 80%. By comparing the results of the cold rolled and annealed tube, it can be found that no recrystallization occurs in the grain of the tube after vacuum annealing at 490 °C/3 h. At this temperature, only the internal stress of the tube is eliminated. Therefore, annealing tube is only selected for subsequent texture research.

### 3.2. Texture Evolution

For Ti-3Al-2.5V alloy tube, different deformation processes will affect the C-axis orientation of the grain basal plane. When the C-axis is mainly concentrated along the radial direction of the tube, the tube has a strong radial texture; when the C-axis is mainly concentrated along the circumferential direction of the tube, the tube has a strong circumferential texture.

**Figure 3:**
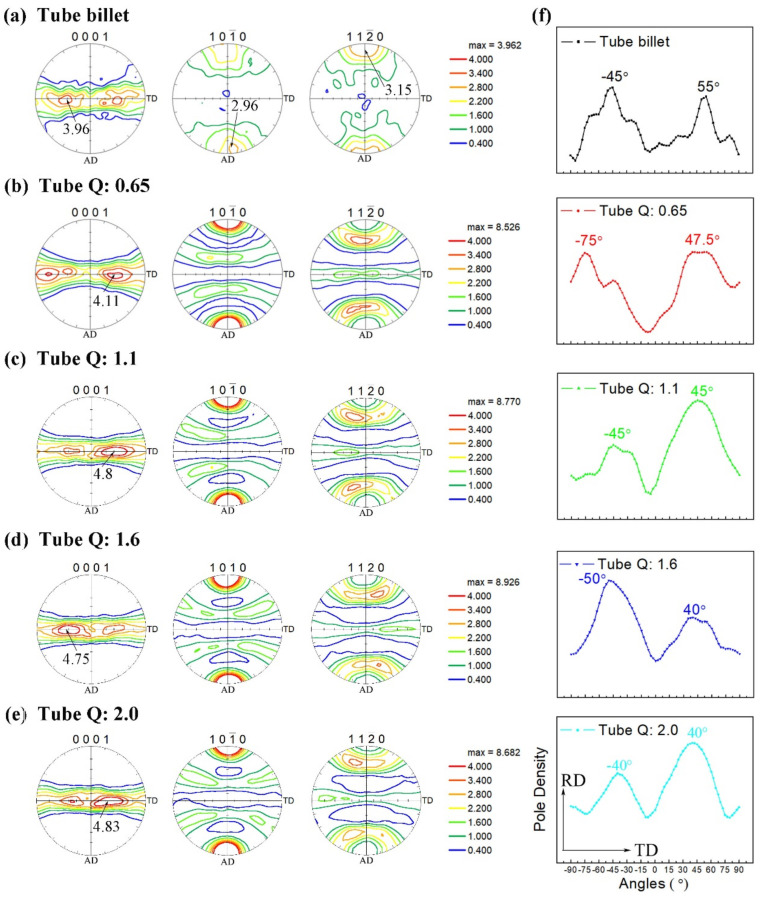
{0001}{1010}{1120} Pole figures and pole density distribution of RD-TD section of rolled tube with different ‘Q’ ratios.

Figure 3 shows the pole figures of {0001}, {1010} and {1120} of the tube billet and rolled tube with different pass ‘Q’ ratios. The pole figures are drawn according to contour lines of the same level, and the contour lines are divided according to the intensity level of {0001} pole figure. Figure 3a is the pole figure of the original tube billet. It can be seen that the pole density of the {0001} pole figure is mainly distributed in the radial (RD) and circumferential (TD) planes of the tube, and the strong pole density values is splitting distributed in the range of ±45° to 60° on both sides of the normal line, and the included angles between the positions of the two maximum points and the normal line are about −45° and 55°. The maximum value of the extreme density is not more than 4.0, indicating that the initial billet has not too strong radial texture. At the same time, it can be seen from the pole figure of {1010} and {1120} that the grain prismatic and pyramidal plane are centrally distributed along the axial direction of the tube. The density value of {1120} pole is stronger reaching 3.15. The {1120} plane is centrally distributed along the axial direction (AD) is the main characteristic of recrystallization texture of α type titanium alloy [28].

The pole figures of {0001}, {1010} and {1120} of tubes rolled with different pass ‘Q’ ratios are shown in Figure 3b–e. It can be seen that for the tube rolled with pass ‘Q’ ratio of 0.65, the pole density of {0001} pole figure is mainly distributed in the range of ±30–90°, and the pole density with strength over 4.0 is mainly distributed in the vicinity of −75° and 35–60°, and the extreme point is located at the Angle of −75° and 50° with RD. It shows that the grain C-axis of tube is mainly distributed in a circumferential direction with circumferential texture. When the tube is rolled with ‘Q’ ratio of 1.1, the part of pole density over 4.0 in {0001} pole figure is mainly distributed in the range of ±15° to 70°, and the angle between the pole point and RD is about ±45°, which is similar to the radial and circumferential uniform distribution. When the tube is rolled with ‘Q’ ratio of 1.6, the pole density of {0001} pole figure is mainly distributed in the range of ±20° to 60°, and the poles deviate from RD about ±45°, indicating that the grain orientation of the tube is gradually concentrated in radial direction, with radial texture characteristics. When the ‘Q’ ratio of the tube is further increased to 2.0, the main distribution range of the pole density of the {0001} pole figure is ±0–60°, and the angle between the pole point and RD is ±40°, and the radial texture is enhanced. At the same time, the {1010} and {1120} pole figures have similar pole density distribution characteristics after rolling with different ‘Q’ ratios. The pole density of{1120} pole figure are distributed around the pole figure in circular shape, while the pole density points of {1010} pole figure are concentrated along the axial direction, and the pole density values are very high. This is the main characteristic of the deformation texture of α titanium alloy with close-packed hexagonal structure. By comparing the {1010} and {1120} pole figure of the original tube billet (Figure 3a), it can be seen that the {1120} oriented grains in this part may have rotated 30° along the C-axis, making the {1010} plane aligned with the axial direction.

**Figure 4:**
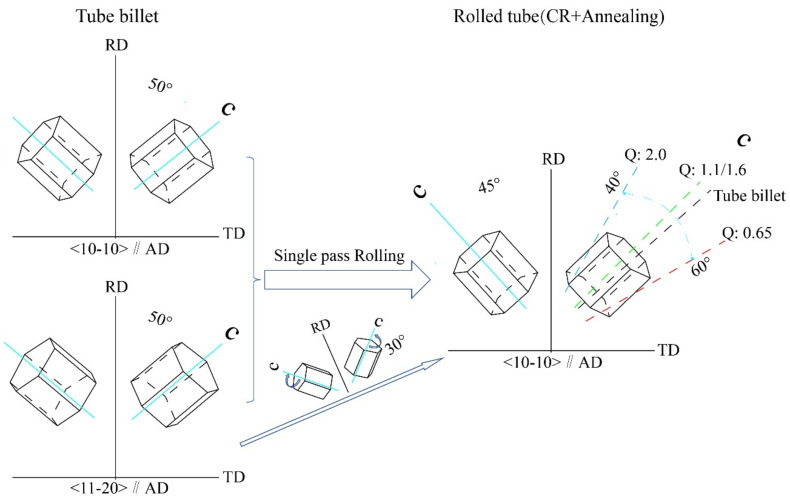
{0001} texture grain evolution diagram.

In order to more clearly and intuitively represent and analyze the variation characteristics of the pole density along the radial-circumferential section of the tube, Figure 3f presents the pole density distribution on the 0° RD-TD section of the {0001} pole figure of the rolled tube with four Q values. It can be seen that the pole density distribution of the RD-TD section of the pole figure presents two peak values, which are distributed on both sides of the RD direction, respectively, representing the concentration points of the two pole density values. The strength of {0001} texture has a certain relationship with the included angle of the peak point of the pole density deviating from the RD direction. Generally, the peak points of pole density are symmetrically distributed on both sides of RD. The degree to which the peak point of pole density deviates from RD can be expressed by the angle between the two peaks. After measurement, the angle between the {0001} peak pole density of tube billet is 100°, and the radial texture of the initial tube is not significant. Similarly, after the tube rolled with ‘Q’ ratio of 0.65, 1.1, 1.6 and 2.0, the included angles between the peak values of {0001} pole density are 122.5°, 90°, 90° and 80°, respectively. It can be seen that, with the increase of ‘Q’ ratio, the peak values of the {0001} pole density of the tube are closer to the RD axis. In addition, when the Q < 1, the angle between the {0001} pole density peak and RD axis of the tube increases, which is not favorable to obtain the radial texture required. Figure 4 shows the evolution characteristics for {0001}{1010}{1120} pole figures and RD-TD section pole density distribution of rolled tube with different ‘Q’ ratios.

**Figure 5:**
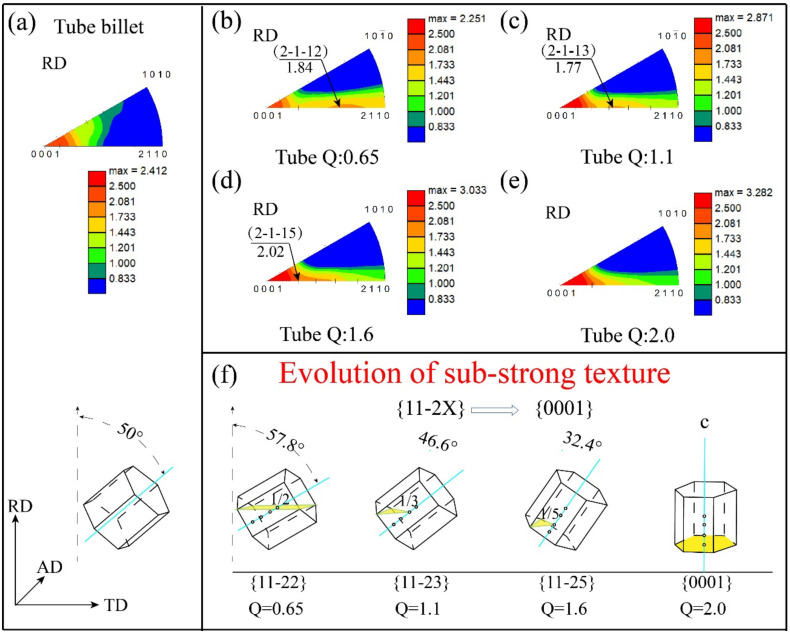
Radial direction inverse pole figures and Grain orientation of sub-strong texture of rolled tubes with different ‘Q’ ratios.

The radial texture is very important for Ti-3Al-2.5V alloy tube. The tube with strong radial texture has strong resistance to radial deformation. The radial inverse pole figure can show the texture distribution along the radial directions. Figure 5 shows the radial direction (RD) inverse pole figure of the experimental tube billet and the rolled tubes with four ‘Q’ ratios. It can be seen from Figure 5a that the original tube billet has a strong {0001} texture along the RD direction of the sample coordinate system, while it cannot be seen that {1010} and {1120} planes are almost concentrated in the radial direction. Figure 5b–e are RD inverse pole figures of tube rolled with four ‘Q’ ratios. The pole density distribution is still dominated by {0001} orientation. Different from the tube billet, the rolled tube with ‘Q’ ratios, except that {0001} plane is oriented in radial direction, There is also a scattered {112X} pole density distribution on the RD inverse pole figure from {0001} to {1120} section, and its concentration decreases with the increase of ‘Q’ ratio.

The change of the radial texture with the rolling ‘Q’ ratio is mainly affected by the orientation distribution of the grain c-axis inside the tube. The more the grains of c-axis deviated to the radial direction of the tube and the smaller the angle with RD, the stronger the radial texture will be. In the RD inverse pole figures of the rolled tube with four ‘Q’ ratios, it is observed that in addition to the strong {0001} texture along the radial plane of the tube, there is also a changing sub-strength texture, whose extreme density intensity is about 1.77–2.02. When the ‘Q’ ratios are 0.65, 1.1 and 1.6, The orientation plane of sub-strong texture is {1122}, {1123}, {1125} in order. When the ‘Q’ ratio is 2.0, no obvious sub-strong texture is found. It indicates that, in the radial plane (AD-TD), except that the {0001} basal plane of most grains is parallel to it, the direction plane of sub-strong texture is {1122}, {1123}, {1125} plane of some grains are parallel to the radial plane. This relationship can be graphically described in Figure 5f. As shown in the figure, when the orientation plane of sub-strong texture is {1122}, the c-axis deviation to RD is the largest, and the deviation angle can be calculated to be about 57.8°; when the orientation plane is {1123}, the c-axis deviation to RD is about 46.6°; when the orientation plane is {1125}, the c-axis deviation to RD is about 32.4°; when Q is 2.0, the sub-strong texture disappears. This is because the X value of {112X} increases sharply with the decrease of the angle between grain c-axis and RD, and the texture changes to the dominant {0001} texture. With the increase of ‘Q’ ratio, more c-axis of grain tends to radial distribution, so the radial texture of tube is continuously enhanced.

### 3.3. Discussion

It is found that the multi-stroke periodic Pilger cold rolling process is a composite deformation process which includes the reduction of tube diameter and wall thickness, and a non-steady and non-uniform plastic deformation process which combines geometry, material and boundary conditions. The stress and strain state in the rolling process dominates the plastic deformation mechanism of tube which is the direct cause of different texture types of tubes. According to the concept of true strain. When the size of tube before and after deformation is known, the total strain of a single pass deformation and the strain components along radial, circumferential and axial directions can be calculated.

**Figure 6:**
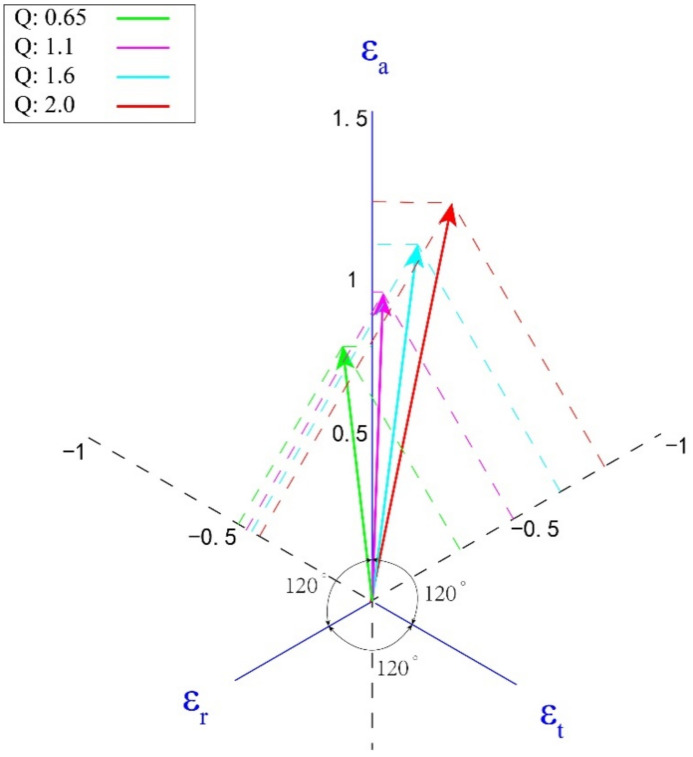
Strain vector in plane triaxial strain diagram.

We calculate the total true strain components in different directions of the rolled tubes with four ‘Q’ ratios in this experiment (Table 3). It can be seen that the rolled tubes with different ‘Q’ ratios have different strain components. Controlling the ‘Q’ ratio and the deformation amount of the rolled passes essentially dominates the distribution of the strain variables along different directions. The radial, circumferential and axial components of the strain can be represented by a vector in a three-axis plane coordinate system with an angle of 120° to each other. This diagram is called the strain diagram, and any change in tube size can be represented by a vector on the strain diagram, called the strain vector. The magnitude and angle of strain vector are directly corresponding to the rolling deformation degree and deformation mode, and the projection length along the three axes represents the deformation assign weights along the radial, circumferential and axial directions [10,27]. The strain diagram of this experiment is shown in Figure 6. Compared with the experimental results, when the strain vector is in the −ε_r_—ε_a_ quadrant, the rolled tube has radial texture, and the more the strain vector is deviated to the −ε_r_ axis, the stronger the radial texture, and the closer to the ε_a_ axis, the weaker the radial texture. Accordingly, in the −ε_t_—ε_a_ quadrant, the rolled tube has circumferential texture, and the strength of texture is related to the degree of strain vector deviation from −ε_t_ axis. It can be seen that the texture evolution of single pass rolled tube is determined by the distribution of the strain.

In the production of Ti-3Al-2.5V tube, ‘Q’ ratio was taken as the main process control parameter, and ‘Q’ ratio was often used to control the degree of circumferential and radial pass deformation in the cold rolling process [7]. ‘Q’ ratio of tube rolling refers to the logarithmic ratio of wall reduction rate and diameter reduction rate.

(1) $$\begin{array}{c} Q=\frac{\ln\left(t_{f}/t_{0}\right)}{\ln\left(D_{f}/D_{0}\right)} \end{array}$$

$t_{f}$ is the wall thickness of finished tube (mm); $t_{0}$ is wall thickness of tube at last intermediate annealing (mm); $D_{f}$ is middle diameter of finished tube (mm); $D_{0}$ is middle diameter of tube at last intermediate annealing (mm).

**Figure 7:**
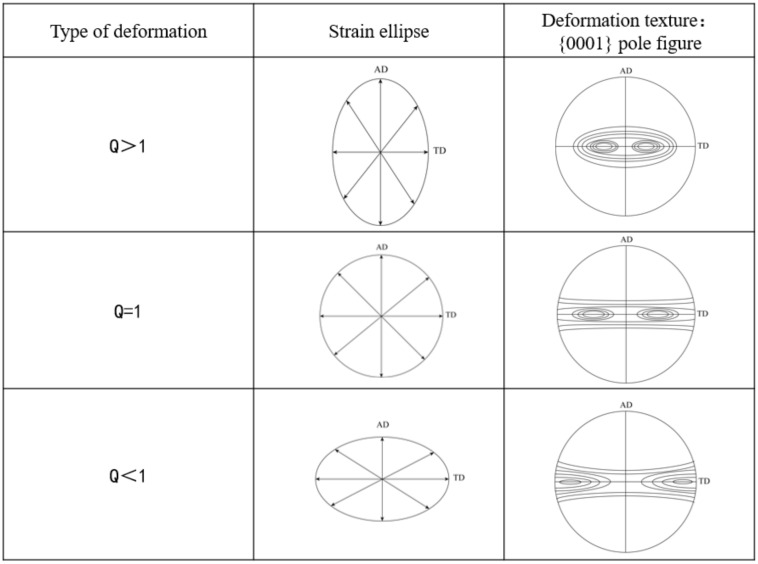
Effect of rolling mode on texture of tube.

According to the formula, log value of wall reduction rate is radial true strain and log value of diameter reduction rate is circumferential true strain. ‘Q’ ratio is a parameter reflecting the relative amount of radial true strain and circumferential true strain. Thus, ‘Q’ ratio mainly controls the radial and circumferential strain of tube deformation. The true radial and circumferential strains can be described by a plane strain ellipse perpendicular to the axial direction of the tube [7,9], as shown in Figure 7. In the rolling process of tube, both radial and circumferential strains are compressive strains. For the deformation with high ‘Q’ ratio (Q > 1), the radial true strain of tube is greater than the circumferential true strain, and the tube will acquire the radial {0001} texture whose normal line of the basal plane is nearly parallel to the radial. For Q = 1, the radial true strain of the deformed tube is equivalent to the circumferential true strain, and the {0001} basal is evenly distributed in the radial-circumferential plane. For Q < 1, the radial true strain of the deformed tube is smaller than the circumferential true strain, resulting in the basal parallel to the circumferential direction and the formation of {0001} texture with circumferential alignment.

Due to the special requirements of aircraft hydraulic systems, the optimal orientation of Ti-3Al-2.5V tube is the basal texture distribution along the radial direction of tube. Through the discussion of the experimental results, the high ‘Q’ ratio rolling can effectively increase the radial strain and reduce the circumferential strain. This strain mode can help the grain basal pole orient along the radial direction, so as to obtain the strong radial texture. Moreover, each ‘Q’ ratio corresponds to a particular dies in Pilger rolling, which makes it difficult to do much research. The understanding of strain vector can not only explain the relationship between ‘Q’ ratio and texture, but also predict other ‘Q’ ratio. Therefore, it is of great practical significance. It can be seen from the research that when the initial texture of the tube is radial-circumferential equally distributed, in order to obtain the radial texture of the tube, the ‘Q’ ratio has to be greater than 2.0, which can provide reference for the production process.

## 4. Conclusions

(1) Ti-3Al-2.5V alloy tubes rolled with different pass ‘Q’ ratios, the {1120} oriented grains in the original tube billet are rotated 30° along the C-axis, so that the {1010} plane in the pole diagram is aligned with the axial direction. With the increase of ‘Q’ ratio, the {0001} peak pole density of tube is closer to RD axis, indicating that the radial texture of {0001} basal plane is gradually enhanced. However, when ‘Q’ ratio < 1, the angle between the peak density point of {0001} pole figure of tube and RD axis will increase, which is not favorable to obtain the desired radial texture.

(2) As can be seen from the radial inverse pole figures, with the increase of the rolling ‘Q’ ratio, more and more grains change from the orientation of {112X} pyramidal to the orientation of {0001} basal plane.

(3) The different ‘Q’ ratio leads to different strain components in radial, circumferential and axial direction, thus affecting the orientation and distribution of grain during the deformation process. The high ‘Q’ ratio rolling can increase the radial strain, which can help the grain basal pole orient along the radial direction of the tube. As a conclusion, when the initial texture of the tube is radial-circumferential equally distributed, it is necessary to select rolling process with ‘Q’ > 2.0 to obtain the ideal radial texture of the tube.

## Figures and Tables

**Table 1:** Chemical composition of Ti-3Al-2.5V titanium alloy ingot.

Element	Ti	Al	V	Fe	O	N	C
wt%	Bal.	3.1	2.5	0.16	0.08	0.01	0.01

**Table 2:** Tube sizes, rolling deformation amount ε and ‘Q’ ratio.

Process Number	Billet Sizes (mm)	Tube Sizes (mm)	Deformation Amount (%)	‘Q’ Ratio
1	Φ25 × 2.8	Φ16 × 2.1	53.8	0.65
2	Φ25 × 2.8	Φ16 × 1.8	60.5	1.1
3	Φ25 × 2.8	Φ16 × 1.5	66.5	1.6
4	Φ25 × 2.8	Φ16 × 1.2	70.4	2.0

**Table 3:** The strain of Ti-3Al-2.5V tube rolling pass.

‘Q’ Ratio	ε_t_	ε_r_	ε_l_
0.65	−0.306	−0.466	0.772
1.1	−0.492	−0.438	0.930
1.6	−0.676	−0.417	1.093
2.0	−0.812	−0.406	1.218

## Data Availability

The data presented in this study are available on request from the corresponding author.
